# Individual changes in neuromuscular performance in the Spanish women’s national football team throughout the 2023 World Cup

**DOI:** 10.5114/biolsport.2025.146789

**Published:** 2025-01-20

**Authors:** Blanca Romero-Moraleda, Ismel Mazola, Aldo A Vasquez-Bonilla, Jaime González-García

**Affiliations:** 1 Department of Physical Education, Sport and Human Movement, Autonomous University of Madrid, Madrid, Spain; 2 Royal Spanish Football Federation, Las Rozas, 28232 Madrid, Spain; 3 Sports Sciences Faculty, University of Extremadura, Caceres, Spain; 4 Exercise and Sport Sciences, Faculty of Health Science, Universidad Francisco de Vitoria, 28223 Pozuelo, Spain

**Keywords:** Female, Soccer, Workload, Monitoring, Jump, Squat, Elite

## Abstract

The aim of this study was to track the neuromuscular performance of the Spanish national women’s football team during the 2023 World Cup. Twenty elite women’s football players were assessed four times during the preparatory and competitive periods. Mean and individual changes in countermovement jump (CMJ) metrics and estimated one-repetition maximum (1RM) for back squat (BS) and hip thrust (HT) exercises were tracked. External and differential internal loads were calculated for field sessions. Strength training load was also monitored using the formula: sets × repetitions × weight × RPE. One-way ANOVA, effect sizes, and individual response analysis were applied. Significant increases in jump height (p = 0.007; ES = 0.12 to 0.44) and concentric propulsive impulse (p = 0.003; ES = 0.15 to 0.47) were observed in MC9 compared to the start of the training camp (MC1). The estimated 1RM in BS was greater in MC9 compared to MC1 (p < 0.001; ES = 1.26 to 2.13), MC4 (p = 0.016; ES = 0.33 to 1.48) and MC6 (p = 0.008; ES = 1.08 to 2.44). Estimated 1RM in HT was greater in MC9 compared to MC1 (p = 0.047; ES = 0.31 to 1.57) and MC4 (p = 0.015; ES = 0.64 to 1.75). Individual analysis showed a positive response in 83% of players in jump height and BS, and in 66% in HT. The Spanish women’s football team showed improved neuromuscular performance throughout the competitive period for the FIFA Women’s 2023 World Cup, especially in the latest stage of the tournament. These results provide insights into the evolution of neuromuscular performance during tournament schedules and highlight the sensitivity of neuromuscular performance monitoring.

## INTRODUCTION

The FIFA Women’s World Cup held in Australia and New Zealand in 2023 represented the largest event in the history of women’s football, marking a significant milestone for the sport. Notably, significant attention is drawn to the evolution of physical performance [1–3]. However, few studies still document the physical capabilities of elite women’s teams. Particularly, when competing in international national football, the majority of sessions are dedicated to player recovery and activation for upcoming matches [4, 5]. It is important to consider that in international competitions, players are exposed to training and match demands that differ from their club routines [6], which may lead to high variability in neuromuscular responses between players. Additionally, external factors such as the menstrual cycle and time zone adaptation can further influence training load, neuromuscular performance and perceptual responses [7, 8]. Therefore, transition periods from a club to a national team require simple and effective load monitoring to guide training strategies and optimize recovery within each microcycle in elite players [9, 10]. However, before and during international tournaments such as the FIFA Women’s World Cup, it is assumed that more time available and heightened supervision from coaching staff will allow individualized training loads of the players throughout the preparatory and competitive period and show superior results in terms of their physical capacities.

In this sense, monitoring neuromuscular performance through actions involving muscular power, such as jumping, sprinting, or over-coming loads at different speeds, is frequently employed as a periodic tool to assess stress-response and training adaptations in team sports such as women’s football [11]. This is key because these activities have direct applicability to on-field performance in numerous high-intensity actions, including linear sprints, changes of direction, kicks, tackles, accelerations, and decelerations, all of which demand rapid contractions and are necessary for the actions of success in football [12]. However, the time available to generate force during these actions is limited. Consequently, muscle strength and force and, particularly, the ability to swiftly produce force, emerge as critical components in optimizing the physical performance of women footballers [13].

To assess neuromuscular performance, the countermovement jump (CMJ) has emerged as a key tool for assessing neuromuscular status in elite sports due to its established reliability [14]. Widely recognized as the gold standard for tracking neuromuscular adaptations in high-performance environments and well established in female football [15], the CMJ test demonstrates excellent repeatability and high sensitivity in measuring fatigue [15]. Likewise, hip thrust (HT) and back squat (BS) exercises are commonly employed to enhance lower extremity strength in women’s football [11]. Furthermore, these exercises exhibit transferability to vertical jump and sprinting actions [16, 17]. Recent research highlighted the efficacy of a 7-week resistance training programme, incorporating HT or BS, in enhancing performance among elite adolescent female footballers [11]. These findings underscore the significance of muscle strength in both HT and BS exercises for the development of peak sports performance in women footballers. Moreover, to estimate muscle strength in practical settings, the load-velocity (L-V) profile has been proposed as a simple and reliable method. This profile allows for the estimation of one-repetition maximum (1RM) based on bar velocity during resistance exercises, serving as an indirect indicator of muscular strength [18]. This methodology has demonstrated accuracy, reliability, and sensitivity in detecting fatigue and adaptations in programming training for football players [19]. Also, the L-V profile is a valid and less invasive alternative to direct testing (e.g. maximal loads, maximal sprints) that could increase fatigue and injury risk during high-demand training [20]. Taken collectively, the longitudinal assessment of the CMJ and the L-V profile in the HT and BS exercises can provide valuable insights into the evolving neuromuscular profiles of female footballers over time.

Accordingly, to optimize overall performance and achieve peak neuromuscular performance, a strategic planning approach is often proposed that includes periods of load intensification followed by a gradual reduction in training load [21]. To achieve this goal, it is essential to create a comprehensive, flexible, and individualized training structure that aligns training goals with athletes’ individual recovery periods [22]. This structure will be based on continuous monitoring of external and internal loads [15], as well as the athlete’s ability to tolerate and recover from training stimuli, resulting in undulating patterns in training load [4]. Properly implementing this stimulus, response, and adaptation process will make it possible to maximize training adaptations and reduce the risk of injury and illness [23]. This form of programming and load control enables progressive decreases in training load, which in turn allows for an increase in the physical determinants in football at opportune times of the season while reducing the risk of overtraining [24]. Thus, we hypothesize that the Spanish women’s national football team would show progressive enhanced neuromuscular performance in CMJ, BS and HT in the last microcycles (MC) compared to the MC in preparation for competing in the FIFA Women’s World Cup. The aim of this study was i) to evaluate neuromuscular performance through the measurement of CMJ kinetic variables, the estimation of 1RM in BS and HT and ii) to describe the TL distribution in elite female footballers during the FIFA Women’s World Cup Australia-New Zealand 2023.

## MATERIALS AND METHODS

### Design

**FIG. 1:**
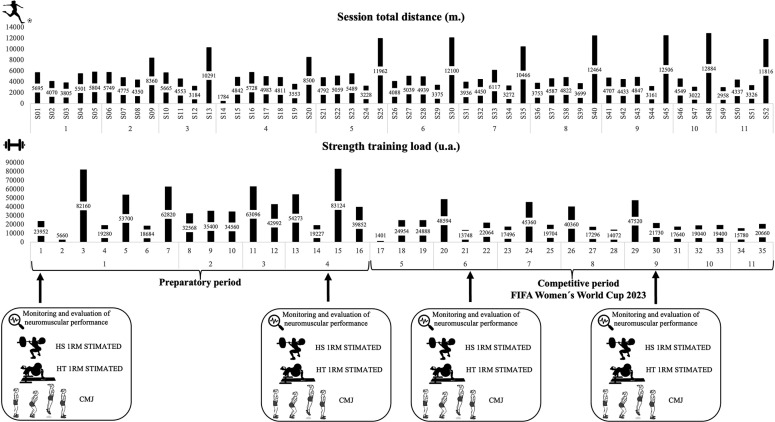
Overall schedule, microcycle workload and neuromuscular performance assessments during the preparatory and competitive period of FIFA Women’s World Cup 2023.

This longitudinal study was performed to track the neuromuscular performance (i.e. CMJ kinetics variables, 1RM estimated for BS and HT exercises) throughout the FIFA Women’s World Cup Australia-New Zealand 2023. The assessment schedule was as follows (Figure 1): the first measurement took place on the first day of the preparatory period (week 1, MC 1); the second measurement was conducted 10 days before the first official match of the FIFA Women’s World Cup (week 4, MC 4); the third measurement coincided with the beginning of the competitive period (week 6, MC 6); and the final measurement was taken twelve days before the last match (week 8, MC 9). Testing was integrated into their routine physical training for evaluation and individual monitoring. The testing procedures adhered to the schedule established by the national team’s coaching staff. All testing sessions were performed before the pitch football sessions during strength training. The tests performed to analyse variations in the neuromuscular performance of the national team included the CMJ test and measuring the barbell mean propulsive velocity at two loads in the BS and HT exercises. Before performing the tests, players completed a standardized 10-min warm-up (including 5 min on a static bike, mobility and stability exercises) followed by one approximation set with approximately 30% of the subjective estimated 1RM for each exercise. The warm-up was guided and supervised by the strength and conditioning coach of the national team.

The average number of microcycle training sessions and strength training programmes completed by the national football players during both the preparatory and competitive phases are presented in Table 1 and illustrated in Figure 1. During the study, the technical staff of the Spanish national team monitored the duration of menstrual cycles and assessed the urinary peak of luteinizing hormone, which was conducted to confirm ovulation, levels of pain/discomfort, nutritional intake, and sleep habits of the footballers.

**TABLE 1:** Overview of the microcycles, on field and strength workload during the World Cup and the previous training camp.

Micro-cycle	Duration of the microcycle (days)	Field sessions in the microcycle (n)	Matches in the microcycle (n)	Gym sessions in the microcycle (n)	sRPE Leg		sRPE Breath		sRPE Cog		Gym TL (Sets × reps × Kg × RPE)	

					Mean	SD	Mean	SD	Mean	SD	Mean	SD
1	5	5	0	7	2321.9	337.9	2163.3	484.7	2169.0	325.6	266256	28223
2	4	3	1 FM vs Panama	3	1747.4	340.3	1659.7	377.4	1742.0	360.2	102528	1455
3	4	3	1 FM vs Denmark	2	1668.0	479.0	1613.4	554.7	1683.5	510.7	106088	14216
4	7	6	1 FM vs Vietnam	4	2425.9	626.1	2246.9	688.0	2382.5	633.0	196476	26847
5	5	4	1 WC vs Costa Rica	3	1984.7	415.4	1923.6	525.2	1977.4	458.5	51243	13579
6	5	4	1 WC vs Zambia	3	1363.3	463.0	1328.5	528.1	964.5	294.6	84406	18199
7	5	4	1 WC vs Japan	3	1292.0	468.2	1374.7	547.6	1333.1	434.6	82560	15489
8	5	4	1 WC vs Switzerland	3	1278.0	413.5	1249.9	492.8	1224.0	366.7	71728	14338
9	6	4	1 WC vs Nederlands	3	1589.1	406.0	1538.4	498.0	1496.3	380.7	86890	16200
10	4	2	1 WC vs Sweden	2	559.6	177.3	522.3	241.6	519.9	174.7	38440	255
11	5	3	1 WC vs England	2	454.6	127.7	434.1	174.5	442.1	129.1	36440	3451


**Microcycle**	**Microcycle duration (min)**			**Microcycle TD (m)**			**Microcycle HSRD (m)**			**Microcycle Sprint Distance (m)**		

	**Mean**	**SD**	**Differences with microcycles (p < 0.05)**	**Mean**	**SD**	**Differences with microcycles (p < 0.05)**	**Mean**	**SD**	**Differences with microcycles (p < 0.05)**	**Mean**	**SD**	**Differences with microcycles (p < 0.05)**
1	334.6	18.9	3.8.10.11	24683.1		3.10.11	1931.9	448.9	2.7.10.11		271.2	4
2	294.2	54.3	10.11	20991.0		10.11	1288.9	459.1	5.6		246.4	3,4,5,6
3	272.9	42.3	10.11	20241.8		10	1794.4	476.0	10		329.6	4,7,10,11
4	501.6	21.6	All	34505.1		All	2760.1	566.7	All		383.8	All
5	322.2	57.8	8	24989.1		10.11	2013.0	690.4	7.10.11		357.3	7,10
6	305.1	40.7	10.11	22729.9		10.11	2073.0	586.3	7.10.11		333.8	7,10,11
7	299.9	84.9	10.11	21906.6		10.11	1246.0	549.7			272.6	
8	265.7	54.9	10.11	20478.8		10	1466.0	633.1			310.7	
9	296.6	48.2	10.11	22582.8		10.11	1715.9	661.5			313.1	
10	178.2	53.9		13539.7			1164.4	599.9			291.7	
11	196.8	39.1		15763.2			1222.3	575.1			331.5	

Note: FM = friendly match; WC = World Cup match

### Participants

Twenty elite women football players (age: 24.9 ± 3.45; body mass (kg): 62.14 ± 6.4; height (cm): 167.5 ± 5.8; lean mass (kg) 29.3.9 ± 3.02 and body fat percentage: 16.1 ± 3.18) of the senior national team of Spain were assessed throughout this longitudinal study. The essential requirement for considering these twenty-three players in this study was that they had been selected to play in the FIFA Women’s World Cup Australia-New Zealand 2023. The exclusion criterion was having suffered a severe injury or illness that caused an evident decrease in performance during the competition. All the players were fully familiarized with the procedures due to the testing protocol being conducted routinely during all FIFA windows throughout the season 22–23. Therefore, this procedure was not invasive for the players at any time. Players were categorized as defenders (7), midfielders (7), or attackers (6) according to field position during matches. All players consented to their participation in the study and data collection and signed an informed consent form on behalf of their national team. All procedures were approved by the Human Ethics Committee of Universidad Autónoma de Madrid (CEI-124-2528), in accordance with the Declaration of Helsinki.

### Training load monitoring

To describe the external and internal load of pitch and strength sessions during the preparatory and competitive periods, total distance (m) (TD), high speed running distance (m) (HSR; i.e., > 18 km · h^−1^) and sprint distance (i.e., > 21 km · h^−1^) (m) [25] covered per microcycle and strength training load are presented (Table 1). Total distance for training and matches were collected using a GPS WIMU PRO device (RealtrackSystems S.L., Almeria, Spain). Intra- and interunit reliability was acceptable (intra-class correlation coefficient value was 0.65 for the x-coordinate, and 0.85 for the y-coordinate) for the systems analysed [26]. The data collected were analysed using the SPRO Software (version 958; RealtrackSystems, Almeria Spain). The average TD covered by all players is presented in Figure 1. For training sessions players were monitored for the entire session, while for matches they were monitored from the start of the match until the final whistle. Both training and match sessions included an on-pitch warm-up.

The internal player workload was monitored using session rating of perceived exertion (sRPE) for each scale, sRPE_breath_, sRPE_leg_ and sRPE_cog_, calculated by multiplying each RPE value by the session duration (in minutes) for each training session (including recovery periods between exercises) and match (including warm-up). The 10-point RPE Borg scale was applied 5–30 minutes after training sessions and matches [27]. The sRPE is a valid indicator of global internal load in football [28]. All players were familiarized with the RPE_breath_, RPE_leg_ and RPE_cog_.

Strength training load was monitored using the following equation [29]: *Strength training load* = *number of sets × number of repetitions × weight lifted (kg) × RPE.* All players reported the RPE score for each exercise after the session [30]. The load in arbitrary units and number of strength sessions are reported in Table 1.

### Procedures

The tests were performed by the national team’s staff, who are experts in this field. These evaluations are part of their regular working methodology, so all the players were fully familiarized with the procedures.

### Countermovement jump

To assess the variation in neuromuscular performance during the preparatory and competitive periods of the FIFA Women’s World Cup, jump metrics were measured using a Hawkin Dynamics Inc. (Westbrook, Maine, USA) dual Force-Platform at a sampling rate of 1,000 Hz. Each participant performed two CMJ and the mean jump [31] was introduced for statistical analysis. The participants stepped onto the force plates and stood completely upright (with extended hips and knees) and remained quiet for at least one second before [32] performing three maximal effort trials following the “3, 2, 1, jump” command. Each participant’s body weight was calculated by averaging the vertical force trace over the first one second of data collection. The onset of movement in the CMJ was defined as the 30 ms before the moment the vertical ground reaction force (vGRF) exceeded the mean force plus or minus five times the standard deviation (SD) of the average force calculated during the weighing phase [33]. Take-off was determined as occurring when the vGRF dropped below 5 SDs of the average force during the first 300 ms of flight. Centre-of-mass (COM) velocity was calculated by dividing vertical force (minus body weight) by body mass and then integrating the product using the trapezoid rule. COM displacement was determined by double integrating the vertical force data [34]. Jump metrics were defined as follows: jump height (the highest displacement of the centre of mass calculated from the vertical velocity at take-off), relative propulsive net impulse (the area under the force-time curve in the propulsive phase minus player weight) and countermovement (CM) depth. The CMJ variables showed good to excellent reliability (jump height: ICC = 0.94 [0.85–0.98]; CV% = 2.90 [1.00–4.79]; SEM = 1.1 cm; SWC = 1.9 cm; relative propulsive net impulse: ICC = 0.93 [0.84–0.97]; CV% = 1.48 [0.51–2.44]; SEM = 0.04 Ns/kg; CM depth: ICC = 0.74 [0.44–0.89]; CV% = 7.64 [2.65–12.63]; SEM = 3.16 cm).

The 1RM was estimated from the individualized linear model as the load (kg) associated with a terminal velocity of 0.3 m/s for BS [18] and 0.25 for HT [35] exercises. The practical 2-point approach involved obtaining the absolute loads corresponding to two different ranges of mean propulsive concentric velocity (MPCV). Due to the evaluation being performed during the gym training sessions and to represent two safe loads that demonstrate high power output, two different weights were used for the BS: specifically, 40 kg, representing 37.21 ± 5.33% of 1RM, and 60 kg, representing 55.81 ± 8.00% of 1RM. For the HT, the weights employed were 60 kg representing 46.93 ± 5.22% of 1RM and 80 kg representing 62.57 ± 6.55% of 1RM. A linear encoder (CLTP, Chronojump, Boscosystem, Barcelona, Spain) was used to measure the MPCV (m/s) in both BS and HT. Three attempts were performed with each weight. The players were instructed to perform the concentric phase of the movement as fast as possible in each repetition. In addition, for greater reliability, they were informed that they had to pause for about two seconds in between repetitions.

The reliability of both estimated 1RM for back squat (1RM BS) and hip thrust (1RM HT) were as follows: 1RM BS: ICC = 0.48 [0.07–0.75], CV% = 7.57 [2.75–12.14], SEM = 11.42 kg, SWC = 17.06 kg; 1RM HT: ICC = 0.46 [0.03–0.72], CV% = 4.67 [1.84–7.50], SEM = 7.85 kg, SWC = 17.85 kg.

### Statistical analysis

Statistical analyses were conducted using IBM SPSS Statistics version 26.0 (IBM Corp., Armonk, NY, EE. UU.). The evaluation of ICC with 95% confidence intervals (95% CI) followed these criteria: poor reliability (< 0.5), moderate reliability (0.5–0.75), good reliability (0.75–0.90), and excellent reliability (> 0.90). Additionally, the standard error of measurement (SEM) and CV% were calculated to identify absolute reliability [36]. Data were analysed using linear mixed models with 95%CI to evaluate the impact of training and competition during both the preparatory and competitive periods of the FIFA Women’s World Cup on neuromuscular performance. In this study, competitive participation, based on total minutes of competition (under/over 302 minutes), was treated as a fixed effect. One-way ANOVA was carried out to compare the external load along the microcycles of the observed period, including preparatory and competitive microcycles. For both analyses, pairwise comparisons were examined using Bonferroni’s post-hoc test. Furthermore, effect sizes (Cohen’s d) were computed between conditions and categorized as follows: ≤ 0.2 (trivial), ≥ 0.2–0.6 (small), ≥ 0.6–1.2 (moderate), ≥ 1.2–2.0 (large), and ≥ 2 (very large) [37]. Individual player response was determined by comparing the change in each dependent variable to its respective SEM. A positive response was defined as a change greater than the SEM, indicating an improvement. A non-response occurred when the change was within the SEM range, suggesting no change. A negative response was defined as a change less than the negative SEM, indicating a decline. Individual analysis results were reported as the percentage of positive responses, non-responses, and negative responses out of the total number of observations (% positive responses/% non-responses/% negative responses). Results are presented as mean ± SD, and the significance level was set at p < 0.05.

## RESULTS

There was a main effect of time on jump height (F_2.080,38.83_ = 4.297; p = 0.019) (F_2.243,41.86_ = 4.701; p = 0.012), and estimated 1RM of BS (F_2.164,24.53_ = 13.07; p < 0.001) and HT (F_1.229,14.75_ = 8.421; p = 0.008). The CM depth was similar across the four testing sessions (F_2.555,47.70_ = 2.684; p = 0.066). According to the external load analysis, a main effect was observed for the variables duration (F_10,205_ = 53.446; p < 0.001), total distance covered (F_10,205_ = 24.699; p < 0.001), HSR (F_10,205_ = 13.997; p < 0.001), and sprint distance (F_10,205_ = 15.484; p < 0.001) throughout the microcycles. The external workload descriptive data and pairwise comparison are displayed in Table 1.

**FIG. 2:**
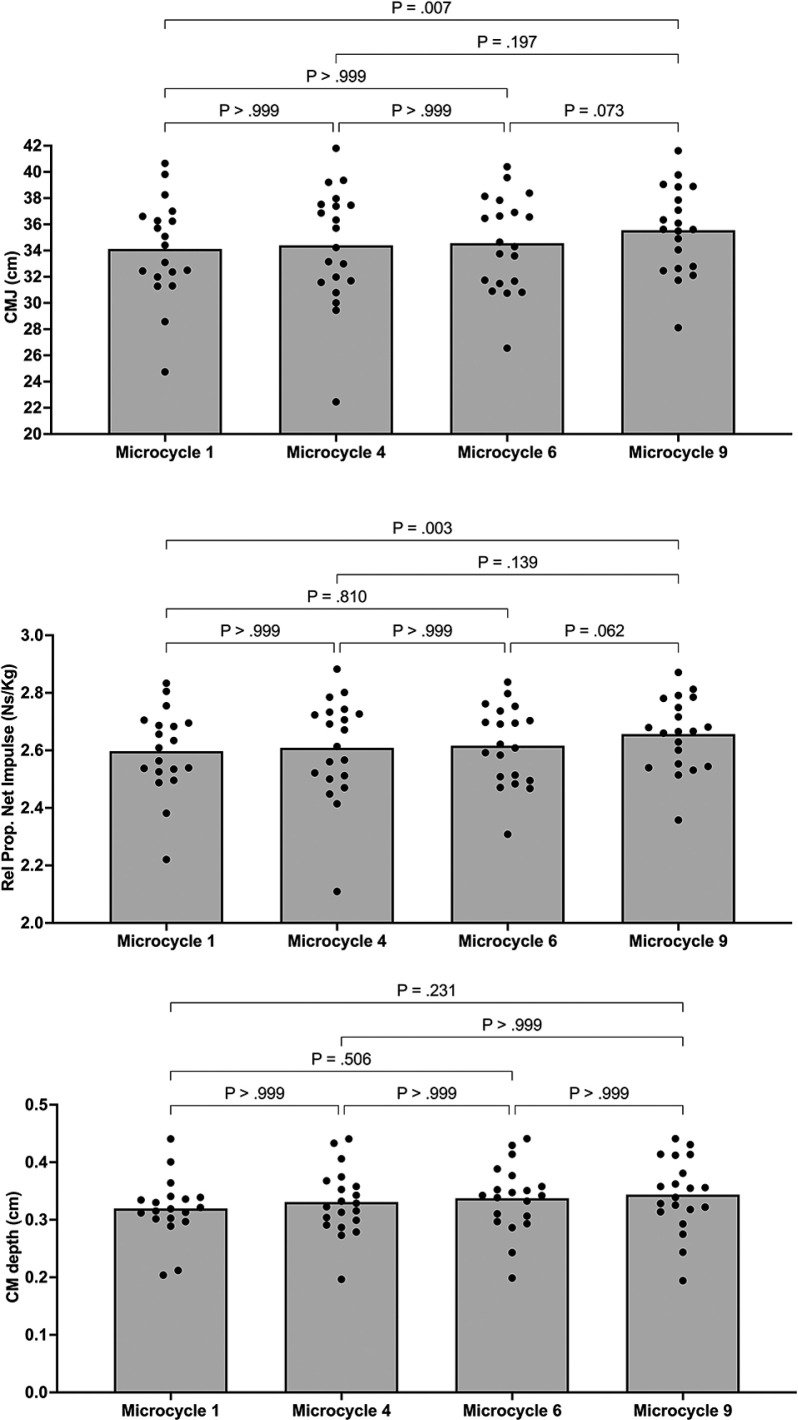
Mean and individual responses in estimated CMJ height, relative propulsive net impulse, and CM depth over the different assessments

The players exhibited a higher jump height (p = 0.007; mean difference = 1.43 cm; ES 90%CI = 0.28 [0.12–0.44]; individual analysis: 83.33/16.66/0) and a higher relative propulsive net impulse (p = 0.003; mean difference = 0.059 Ns/kg; ES 90%CI = 0.31 [0.15–0.47]; individual analysis: 50/45/5) in the evaluation of microcycle 9 compared to the evaluation of microcycle 1. There was also a trend for both jump height and relative propulsive net impulse to be significantly higher between the evaluations of microcycles 6 and 9, showing a small effect size between the two evaluations (ES = 0.27 to 0.28). All pairwise comparisons are displayed in Figure 2.

**FIG. 3:**
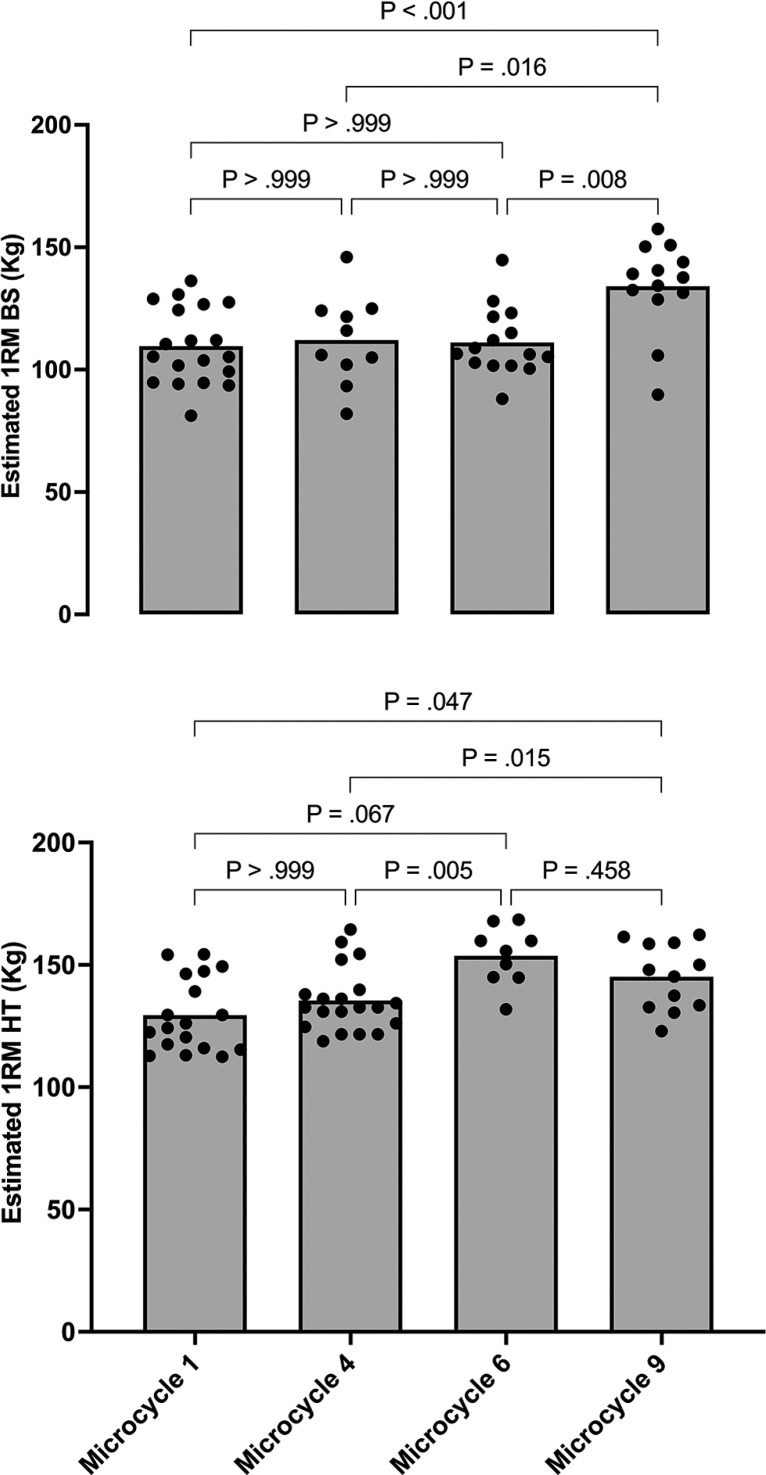
Mean and individual responses in estimated 1RM for back squat and hip thrust over the different assessments

The estimated 1RM BS values were higher in microcycle 9 compared to the other three evaluations (p < 0.016; mean difference = 21.93 to 25.44 kg), showing ES 90%CI = 1.70 [1.26–2.13], 0.91 [0.34–1.48], and 1.76 [1.08–2.44], individual analysis of 83.3/16.6/0, 72.72/18.18/9.09, 80/20/0 for microcycles 1, 4, and 6, respectively. Moreover, the estimated 1RM HT values were significantly higher in the evaluation of microcycle 6 (p = 0.005; mean difference = 18.31 kg; ES 90%CI = 1.20 [0.64–1.75]; individual analysis: 66/33/0) and 9 (p = 0.015; mean difference = 9.7 kg; ES 90%CI = 0.97 [0.65–1.29]; individual analysis: 50/41.6/8.33) compared to those presented in microcycle 4 (Figure 3).

## DISCUSSION

The aim of this study was to track the neuromuscular performance of the Spanish national women’s football team, describing the training load distribution during the Australia-New Zealand 2023 World Cup. Overall, these players exhibited significant neuromuscular improvements. The principal findings were: (1) vertical jump height and relative propulsive net impulse variables improved significantly in the last two evaluations (MC6 and MC9) compared to the first evaluation (MC1), while CM depth remained consistent across the four evaluations, (2) BS values were greater in the last evaluation (MC9) compared to the previous three evaluations (MC1, MC4, and MC6), and (3) HT results were higher in the third (MC6) and fourth evaluation (MC9) than in the first evaluation (MC1).

Contemporary international football tournaments are characterized by a dense schedule with a high number of matches played in a short period of time [9]. Consequently, pre-tournament training camps serve to ensure that players are tactically, physically, and mentally prepared for the demands of the tournament. Therefore, a significant challenge for national team staff is managing the players’ physical preparation during the transition from club-tocamp-to-tournament. This is particularly important given the variable prior exposure to different training loads and various leagues in which Spanish players compete, such as Liga F, FA Women’s Super League and Liga MX Femenil. Periodic assessment of the neuromuscular performance during both preparatory and competitive periods allows individualized power-speed-oriented strength training prescription for individual players and facilitates the identification of potential neuromuscular performance impairments whether due to fatigue status or inadequate load assimilation [31]. Therefore, it is crucial to establish a reliable method for neuromuscular assessment to be used during strength training sessions. The primary practical application of the proposed methodology for technical staff is to enable faster decision-making in configuration of training stimuli. Therefore, the dependent variables monitored are related to speed-power strength because most player actions during a football game involve very rapid contractions, limiting the time available to produce force. Hence, strength capacity, and particularly the ability to produce force quickly, is a critical aspect of optimizing physical performance [38].

As in previous research [39], jump metrics for the CMJ test, such as jump height, relative propulsive net impulse, and countermovement depth, were used as main criterion measures of neuromuscular performance and fatigue [40]. However, the expression of fatigue in the CMJ test is not evident in a reduction in jump height, but rather in alterations in the strategy used to generate impulse [41, 42]. The results of the current study showed a progressive increase in jump height and relative propulsive net impulse across the preparatory and competitive periods in 42% and 50% of the analysed players, with greater values for these variables in MC9 compared to MC1, while maintaining jump strategy. Like previous studies [43, 44], the results of the current study confirm that jump height is a sensitive measure of neuromuscular performance and allows us to identify adaptations [45]. Taken collectively, this tool reaffirms itself as a fast, sensitive, and reliable alternative for evaluating the neuromuscular readiness of female soccer players, thus providing objective information to support decision-making by the technical staff. Specifically, other studies have shown that neuromuscular performance, measured via changes in CMJ and V1 load in squat exercise, demonstrated no changes in CMJ height and small to moderate improvements from pre-season to the end of the season in V1 load, with a wide variability in response throughout the season [43], Similarly, our results showed that the estimation of 1RM based on changes in velocity at submaximal loads was significantly higher in microcycle 9 compared to the other cycles (p < 0.016), suggesting an increase in the neuromuscular capacity due to a higher force production, and therefore, acceleration, against a given external load. In these terms, the estimated 1RM of HT was also higher in microcycle 6 (p = 0.005) and microcycle 9 (p = 0.015), with moderate to large effect sizes. The study’s data support the notion of individualized responses to training. Specifically, only 42% of the players showed a progressive increase in jump height, while 50% exhibited improvements in relative propulsive net impulse across the preparatory and competitive periods. Additionally, the variation in estimated 1RM values across microcycles provides further evidence of individual differences. For instance, 1RM estimates were significantly higher in microcycle 9, with moderate to large effect sizes, and also showed marked increases in microcycle 6 (p = 0.005) and microcycle 9 (p = 0.015). These findings reveal distinct patterns of neuromuscular adaptation, suggesting that while some athletes respond robustly to training stimuli, others may experience smaller or delayed improvements. This data-driven insight emphasizes the need for individualized monitoring to optimize training and recovery for each player.

Our findings are consistent with previous studies, which also showed improvements in jump height and movement velocity at submaximal loads during the season [43, 44]. Interestingly, our results show a more pronounced increase towards the end of microcycle 9, while previous studies highlight greater variability throughout the season. This could indicate that the training programme used had a significant impact on improving neuromuscular performance, especially in the final stages. This may be due to the manipulation of training loads during the competitive period compared to the microcycles of the preparatory period (Table 1). According to our results, the total distance covered, HSR, and sprint distance were significantly lower from microcycle 5 (beginning of the competitive period) compared to the end of the preparatory period (microcycles 3 and 4). This reduction in total training load may have allowed for facilitated recovery due to the inverse association between the average load in the last days and the expression of lower limb strength, suggesting that, at times of the season when the weekly training load is reduced, it is associated with the observed increases in neuromuscular performance [46]. Throughout the observation period, various strategies were implemented to enhance recovery, especially in the hours following each competition. In the post-match session, two subgroups were formed based on three items: minutes played (> 60 minutes) [47], impaired wellness and limited perceived recovery, being the subjective metrics recorded during the morning after the match. For the group requiring more intensive recovery, specific strategies were applied, including active recovery, adjustments in the volume and intensity of current and future sessions, as well as physiotherapy and compression therapy. These interventions, along with an adjusted load distribution throughout the tournament, helped improve the players’ recovery, reducing accumulated fatigue and allowing for better adaptation and expression of neuromuscular capacities, thereby decreasing the risk of injury [48].

Since the characteristics and physical demand of international tournaments, where there are fewer rest days among matches than in a national competition, players’ performance may be more affected, as both neuromuscular and physiological responses may need between 3 and 5 days to return to their optimal values [49]. At this point, one might think that as the microcycles with their respective matches went by, the chronic load would increase, producing greater fatigue in the players and therefore decreasing their neuromuscular performance. However, contrary to this expectation, it was found that the players increased their capacity as the weeks went by, reaching peaks of performance in the final phase of the tournament. This result may be related to the total distance at different velocity thresholds and tonnage (AU) load distribution (Table 1 and Figure 1). All these variables show how the load volume fluctuates throughout the whole training camp. It can be observed that the strength workload starts with a high volume of work, with up to seven gym sessions in MC1 and four in MC4. After the completion of MC4, which is the last preparatory phase, it can be seen how from MC5 to MC9 it drops to a maximum of 3 strength sessions. Eventually, in both MC10 and MC11 it drops to 2 strength sessions (Table 1). It should be noted that the microcycles were not all of the same duration, varying from 4 to 7 days. Even so, a decrease in volume can be seen, aimed at improving performance through a tapering strategy [50].

Moreover, due to the multifactorial nature of injury mechanisms and the reductive perspective of attributing injuries to one or a few risk factors, muscle strength levels and load management have been shown to modulate the load-injury relationship [51]. Therefore, during periods of match congestion, such as international tournaments, competing with optimal muscle strength levels is even more crucial. In this regard, the load management strategy (both for field load and strength training) followed a gradual increase in the TL during the pre-World Cup training camp, maintaining that TL between microcycles 6–9 (the group stage of the tournament), and finally reducing it from MC 9 onwards (the final stage of the tournament). This training programming led to gradual increases in estimated strength levels based on the load-velocity relationship from the start of the tournament onwards (Figure 3), which could present a win-win relationship as it could be associated with a lower risk of injury, higher sports performance, faster recovery patterns and higher potentiation effects after acute resistance training sessions in the decisive phase of the competition.

Due to its ecological nature, this study presents a number of limitations that must be considered for its proper interpretation. Firstly, performance assessments (CMJ and L-V profiles) were evaluated in different training locations and under different climatic conditions, as the Spanish national team moved from venue to venue throughout the training camp and during the World Cup. Consequently, environmental variables such as humidity or temperature could not be controlled, which could lead to modifications in the final performance of the physical tests. Another considerable limitation is the use of different absolute external loads (i.e., kg) to estimate 1RM from the load-velocity relationship, which showed poor reliability. As it was during a competitive period, there was no opportunity to address this limitation. The evaluations were carried out during the training sessions themselves, so the training objective determined the load to be used for the calculation of the estimate, which may lead to estimates with lower validity than the gold standard (the direct 1RM test). Future research should analyse other factors such as diet, menstrual cycle, and various recovery strategies that may influence neuromuscular performance in elite women’s football.

## CONCLUSIONS

The findings reveal that the Spanish women’s football team showed improvements in neuromuscular performance throughout the FIFA Women’s World Cup Australia and New Zealand 2023. Notably, increases were observed in CMJ performance and estimated 1RM for BS and HT exercises, particularly in the later stages of the tournament. These observations suggest that neuromuscular capacity can be progressively optimized during an elite-level competition. This research provides valuable insights that can help bridge the gap between sports science and the day-to-day practices of women’s national football teams.
